# Analysis of the Failure and Performance Variation Mechanism of MEMS Suspended Inductors with Auxiliary Pillars under High-g Shock

**DOI:** 10.3390/mi11110957

**Published:** 2020-10-25

**Authors:** Lixin Xu, Yiyuan Li, Jianhua Li

**Affiliations:** 1 School of Mechatronical Engineering, Beijing Institute of Technology, Beijing 100081, China; jhli@bit.edu.cn; 2 Beijing Institute of Control and Electronic Technology, Beijing 100038, China; liyiyuan510@foxmail.com

**Keywords:** microelectromechanical systems (MEMS) suspended inductor, high mechanical shock, MEMS reliability

## Abstract

Microelectromechanical systems (MEMS) suspended inductors have excellent radio frequency (RF) performance and they are compatible with integrated circuit (IC). They will be shocked during manufacturing, transportation, and operation; in some applications, the shock amplitude can be as high as tens of thousands of gravitational acceleration (g, 9.8 m/s^2^). High-g shock will lead to the inductor deformation which affects its performance or even failure of the inductor structure. However, few studies have been carried out on the inductors under high-g shock. In this study, a kind of MEMS suspended inductor with excellent RF and mechanical performance is designed and fabricated. The failure and performance variation mechanism of the inductor under high-g shock is analyzed by measuring and comparing the performance measurement results and the π model parameters extraction results of the inductors before and after air cannon shock test. The results show that the increase of energy loss caused by substrate parasitic effect and the properties variation of the coil material affected by high-g shock are the main reasons for the decrease of RF performance parameters, and the critical stress exceeding the interlayer adhesion is the main reason for the failure of the inductor.

## 1. Introduction

The inductor is one of the main passive components in radio frequency integrated circuits (RFIC). The inductors in traditional RFIC are fabricated by standard integrated circuit (IC) planar process. However, due to the semiconductor characteristics of silicon, which is usually used as the substrate of the inductors, there are serious losses in the inductors on the traditional RFIC, which makes the quality factor (*Q* factor) of the inductors low [1,2,3]. Microelectromechanical systems (MEMS) suspended inductors fabricated by MEMS technology can greatly reduce the loss of inductor by lifting the coil several micrometers above the substrate [4,5,6,7,8], so as to achieve a higher *Q* and meet the requirements of high-performance RFIC for inductors. By optimizing the structure of the inductor, the radio frequency performance of the inductor including Q factor and inductance can be improved. Many studies on inductor optimization have been established nowadays. Zheng et al. designed a 2.7 nH suspended inductor with maximum Q factor 49 at 8.2 GHz. Thick benzocyclobutene (BCB) was employed as the supporting dielectric and a backside cavity was etched and removed to improve the Q factor of the inductor [9]. Li et al. presented a self-packaged high Q factor inductor based on substrate integrated suspended line technology, the substrate was designed hollowed in a specific shape to reduce the substrate loss [10]. Stojanović et al. presented a kind of meander inductor and an accurate method for inductance calculating of the meander inductors [11]. In addition, by reasonably designing the process of MEMS inductors, it can be fully compatible with IC. It has been realized in many reports that MEMS suspended inductors are used to replace traditional IC on-chip inductors to improve circuit performance and to reduce the power consumption of the circuit. For example, they can be used in amplifiers to achieve high gain, wide bandwidth, and low noise [12]; the oscillation circuits with a MEMS suspended inductor can be used in voltage controlled oscillators to significantly reduce oscillator noise [13]; MEMS inductors can be used on conductive chips of radar to improve radar cross section [14]. However, MEMS inductors tend to be affected by various kinds of mechanical shock during fabrication, shipping, and operation. Especially in some extreme application conditions, they tend to withstand high mechanical shock in a very short period of time; the shock amplitude can be as high as 10^4^–10^5^ gravitational acceleration (g, 9.8 m/s^2^) [15].

Copper, a plastic material with high conductivity, is usually selected as the material of MEMS inductor coil to reduce the ohmic loss of the inductor. When the stress of the coil exceeds the tensile strength of copper under shock, the coil will fracture and the device will fail. When the stress exceeds the yield strength of copper but not the tensile strength, the coil will undergo plastic deformation, resulting in the unrecoverable changes of the inductor performance. When the stress does not exceed the yield strength, the coil will undergo elastic deformation, and the inductor performance also varies during shock and deformation process. When the inductor performance varies, it will affect the operation of the circuits. For mechanical performance evaluation and reliability optimization purposes, it is necessary to investigate the performance variation and failure mechanism of MEMS suspended inductors operating in a high-g shock environment.

Most of the studies on MEMS suspended inductors under shock nowadays are through simulation and experiment, the acceleration amplitude is usually as low as tens or hundreds of g. Hoa Thanh Le et al. presented a drop test on the designed MEMS inductors and they measured the inductors after the drop test, but they only qualitatively studied if any visible damage occurs to the inductors and whether they can still work properly after the test [16]. Li et al. simulated the inductance value variation of a MEMS suspended inductor under shock, but the shock acceleration amplitude was only 1 g of gravity acceleration, and the Q factor variation was not considered [17]. Chiu et al. designed an acceleration sensor based on the principle that the resonant frequency will vary after the suspended inductor in the oscillation circuit is deformed under shock. But only low shock amplitude (10 g level) was considered and the relationship between the RF performance of the inductor and shock amplitude was not established [18]. Wu et al. simulated several kinds of MEMS suspended inductors. They applied a mechanical shock with the same amplitude of 450 g on the inductors to obtain the deformation of each inductor under shock, so as to analyze the mechanical properties of each kind of inductor. However, the RF performance variation of the inductors after shock was not considered in this study [19]. The studies above are qualitative studies on whether the structure of the inductor coil deforms or fails after shock, but the influence of shock on the performance of the inductor is not analyzed.

In this investigation, the RF performance variation and failure mechanism of MEMS suspended inductors with auxiliary pillars under high-g shock of which acceleration amplitude is as high as tens of thousands of g are studied, as the inductors without auxiliary pillars under shock with a lower acceleration amplitude (up to 20,000 g) was studied in our former paper [20]. First, MEMS suspended inductors with double auxiliary pillars are designed and fabricated. The theoretical calculation results and the test results show that the MEMS inductor has good mechanical properties and good RF performance. Then air cannon shock tests with high acceleration amplitude are carried out. To conclude whether the inductor samples fail after the shock test, first the inductor samples are placed under a microscope to observe whether they are damaged, such as coil fracture and coil falling off. Then they are measured by an Agilent N5224a (Agilent, Palo Alto, CA, USA) vector network analyzer and a Cascade probe station, the quality factor and the inductance are measured and the values of each π model elements of the shocked inductors are extracted to conclude if the inductors after shock are damaged or intact more accurately. Finally, the experiment results are presented and discussed. The RF performance variation mechanism of the inductors which are not failed after the shock test and the failure mechanism of the failed inductors are analyzed, the yield stress and the critical shock acceleration amplitude of the inductors are also deduced form the test results.

## 2. MEMS Suspended Inductor Sample and Fabrication

To ensure the inductor can resist shock loads with tens of thousands of g, the structure of the inductor needs to be improved. Adding auxiliary pillars is an effective way to improve the mechanical properties of the MEMS suspended inductors. Compared with another common method, adding a supporting layer, of which material is usually silicon, the loss caused by the auxiliary pillars is smaller as the contact area between coil and substrate is smaller, so the influence of the supporting structure on the RF performance of the inductor is smaller [19]. In this study, a 1.5 turns MEMS suspended inductor with double auxiliary pillars is chosen as the inductor sample in order to achieve an excellent mechanical property and to ensure that its RF performance is not significantly affected. Square is chosen as the shape of the inductor coil and the fabrication process of the inductor is mainly considered. The fabrication process of the square shaped inductor is mature, and it is easier to ensure the accuracy of lithography. Compared to the meander inductors, square coil inductors occupy less area with the same inductance.

**Figure 1:**
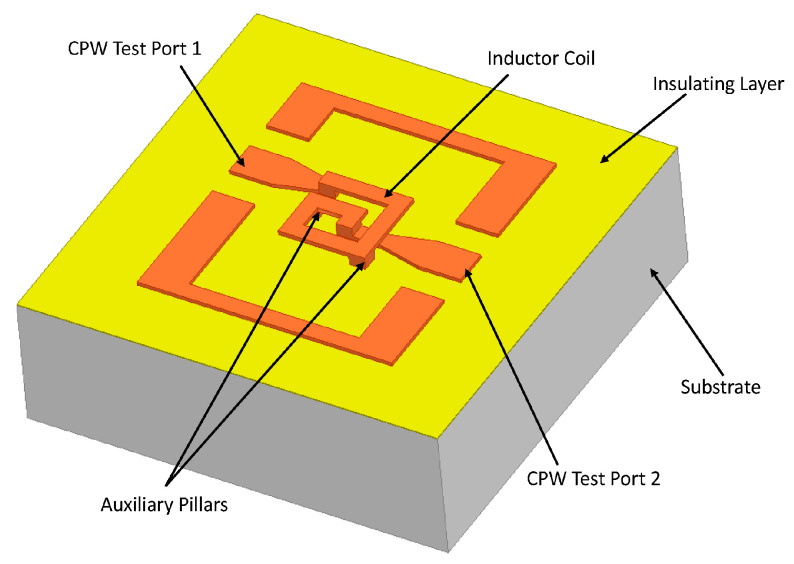
Schematic of the microelectromechanical systems (MEMS) suspended inductor sample.

The mechanical properties of the inductor sample are given priority in the design of the structure parameters. On the basis of ensuring the shock resistance, the feasibility of the fabrication process of the inductor structure is comprehensively considered. The RF performance of the inductor is optimized by the parameter sweep optimetrics function of HFSS (ANSYS HFSS 16.1, ANSYS, Canonsburg, PA, USA) software. The schematic of the MEMS suspended inductor is shown in Figure 1. The inductor coil is made of copper because compared with other conductive material, such as aluminum and gold, the conductivity of copper is higher and the ohmic loss caused by copper coil is less than aluminum or gold coil. Besides high conductivity, copper is also cheaper than gold and silver. The thickness of silicon dioxide insulation layer is 1.5 μm, the thickness of silicon substrate is 1 mm and the substrate resistivity is 10 Ω∙cm. The test ports of inductor are designed as CPW (Coplanar waveguide) ports and the port impedance is designed to be 50 Ω. On the premise of meeting the requirements of probe size of the probe station for test, the design size of the port can be calculated and determined by ADS (Advanced Design System 2016, Agilent, Palo Alto, CA, USA) software.

**Figure 2:**
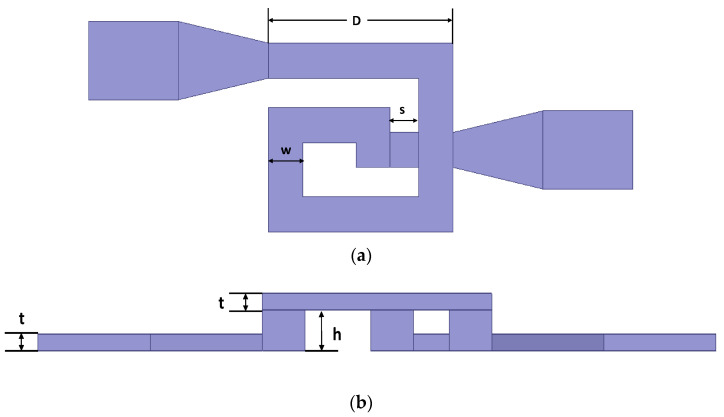
Geometric parameters of the MEMS suspended inductor coil. (**a**) The top view of the inductor coil; (**b**) the side view of the inductor coil.

The geometry parameters of the inductor coil are shown in Figure 2. The outer diameter of the coil is *D* = 200 μm, the number of turns is *n* = 1.5, the spacing is *s* = 40 μm, the wire width of the coil is *w* = 30 μm, the wire thickness is *t* = 10 μm and the suspension height is *h* = 20 μm.

**Figure 3:**
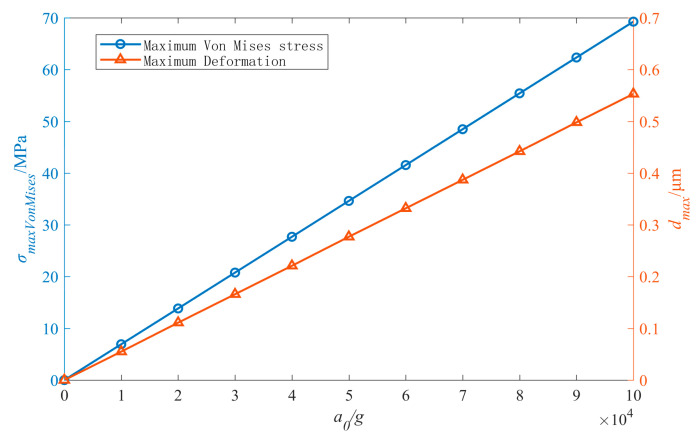
The mechanical response of the inductor sample under shock.

The modal analysis of the inductor structure is carried out with ANSYS workbench software (ANSYS 15.0, ANSYS, Canonsburg, PA, USA), the first three modal frequencies of the inductor are 0.288 MHz, 0.532 MHz, and 1.091 MHz. The shock pulse width generated by the shock test equipment including air cannon and Hopkinson bar ranges from tens to hundreds of microseconds. When the shock duration is comparable to the vibration time period of the coil structure, the structure experienced the shock pulse as a dynamic load and the absolute acceleration response will be amplified to various degrees [21,22]. The mechanical properties of the inductor sample is analyzed with the method in [22]. In order to obtain the mechanical properties of the inductor sample under harsher conditions, the half sine pulse shock loads with duration of 20 μs and amplitude of 10,000–100,000 g are applied to the inductor. The mechanical response including the maximum Von Mises equivalent stress and the maximum deformation of the inductor sample under different shock loads are calculated, as shown in Figure 3.

The calculation results show that when the inductor sample is under mechanical shock with 100,000 g acceleration amplitude, the maximum Von Mises equivalent stress of the coil is 69.26 MPa and the maximum stress occurs at the position where the coil is connected with the pillar of CPW port 1, the maximum deformation of the coil is only 0.553 μm. The results show that the inductor sample has good mechanical properties.

**Figure 4:**
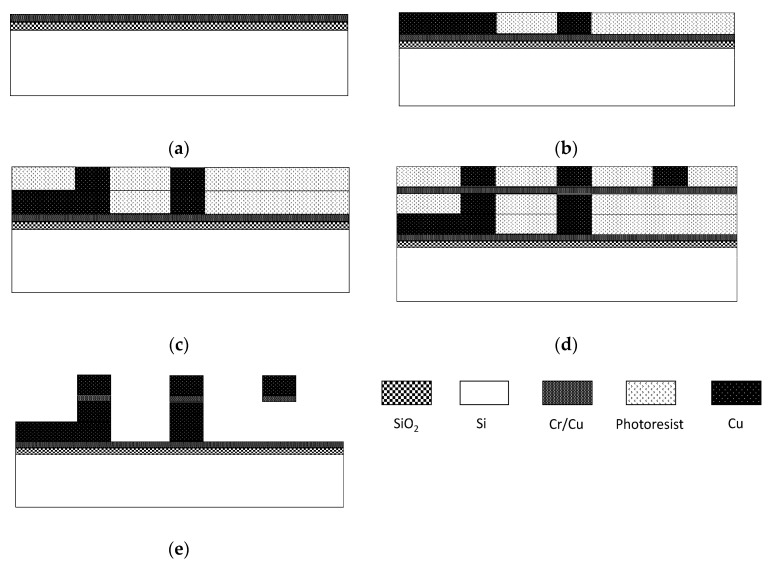
Fabrication process of the MEMS suspended inductor. (**a**) Seed layer deposition; (**b**) photoresist (PR) mold patterning and copper electroplating; (**c**) PR mold patterning and copper electroplating; (**d**) seed layer deposition, PR patterning and copper electroplating; (**e**) PR strip and seed layer etch.

A surface micromachining process based on a positive photoresist is employed to fabricate the MEMS suspended inductors. The fabrication process is shown in Figure 4 and the whole process is illustrated by the following steps:

1. As Figure 4a shows, a 1.5 μm thick silicon oxide insulating layer is first deposited using a plasma enhanced chemical vapor deposition method. Then wash the wafer with mixture of concentrated sulfuric acid and hydrogen peroxide. A chromium/copper (Cr/Cu) seed layer is then deposited on the wafer by a magnetron sputtering process, the thickness of Cr and Cu are 200 Å and 1000 Å respectively.
2. As Figure 4b shows, a 10 μm AZ4620 positive photoresist is spin coated and patterned with the first mask after treating the wafer with Hexamethyldisilazane (HDMS) to remove moisture on it. Then the underpass lines and the test ports are electroplated in the molds; electroplating thickness is 10 μm. A mixture of copper sulfate and sulfuric acid is chosen as the electroplating bath in all electroplating process steps.
3. As Figure 4c shows, a 10 μm AZ4620 photoresist is spin coated and patterned with the second mask. Then, the pillars of the suspended inductor are electroplated.
4. As Figure 4d shows, a Cr/Cu seed layer is deposited on the wafer by magnetron sputtering and the thickness of Cr and Cu are also 200 Å and 1000 Å respectively. Then a 10 μm AZ4903 photoresist is spin coated and patterned with the third mask, the spiral coil of the suspended inductor is finally electroplated.
5. As Figure 4e shows, all photoresist and seed layers are removed in sequence to release the suspended structure. The photoresist is removed by sodium hydroxide and acetone, the remaining acetone is dissolved with anhydrous alcohol, the copper seed layer is removed by mixture of ammonia and hydrogen peroxide, and the chromium seed layer is removed by mixture of potassium ferricyanide and sodium hydroxide.

**Figure 5:**
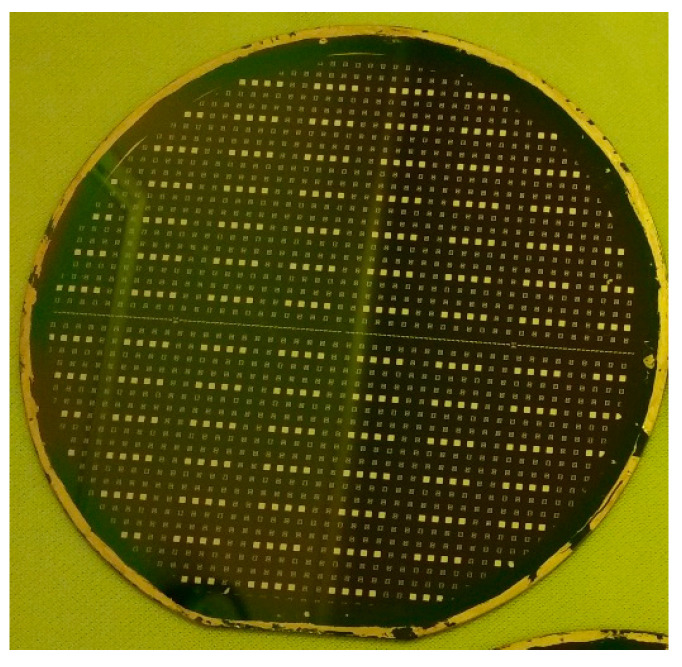
Photo of the MEMS inductor wafer.

**Figure 6:**
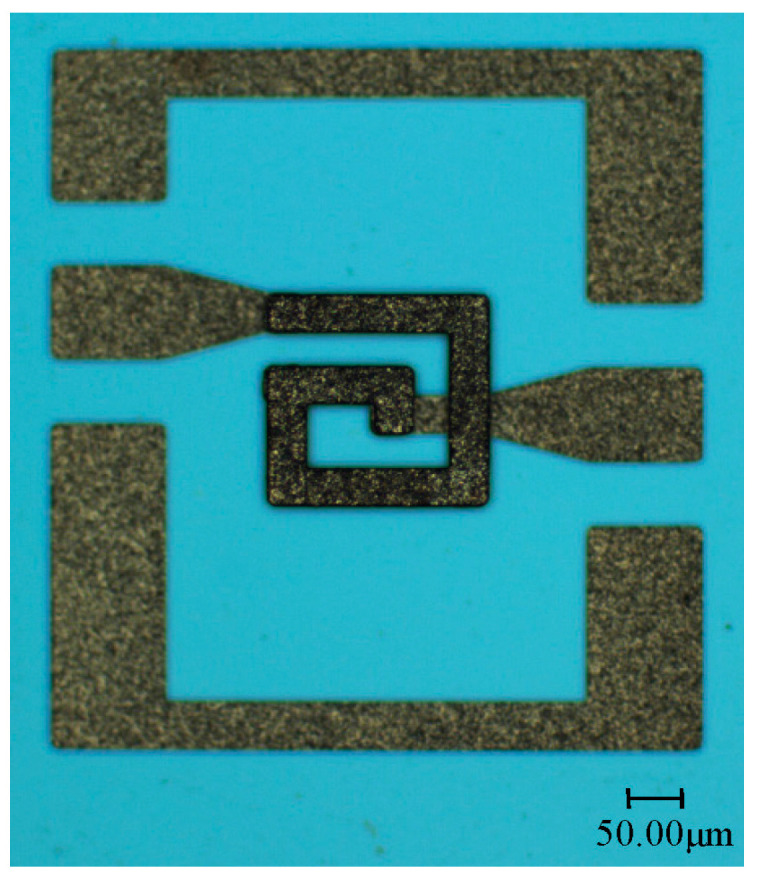
Photo of the MEMS suspended inductor.

The photo of the fabricated MEMS suspended inductor wafer and the photo of the inductor under microscope are shown in Figure 5 and Figure 6, respectively. It can be found that the fabricated inductors have good consistency and good surface quality from Figure 5 and Figure 6.

The RF performance of the fabricated MEMS suspended inductors were measured by an Agilent N5224A (Agilent, Palo Alto, CA, USA) vector network analyzer and a Cascade probe station. S parameters of the inductors were measured by the vector network analyzer and the probe station, then the S parameters were converted to Y parameters. The Q factor and the inductance were extracted with the Y parameters of the inductor [23]:

(1) Q=−Im(Y11)Re(Y11)

(2) L=Im(1Y11)2πf

**Figure 7:**
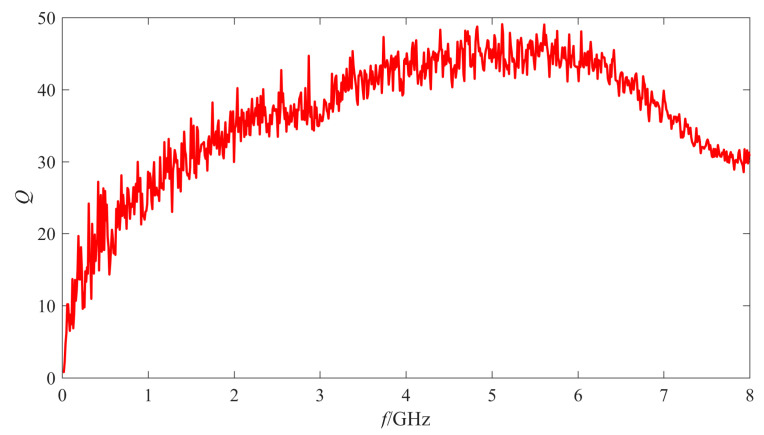
The quality factor (Q factor) of one fabricated MEMS suspended inductor.

**Figure 8:**
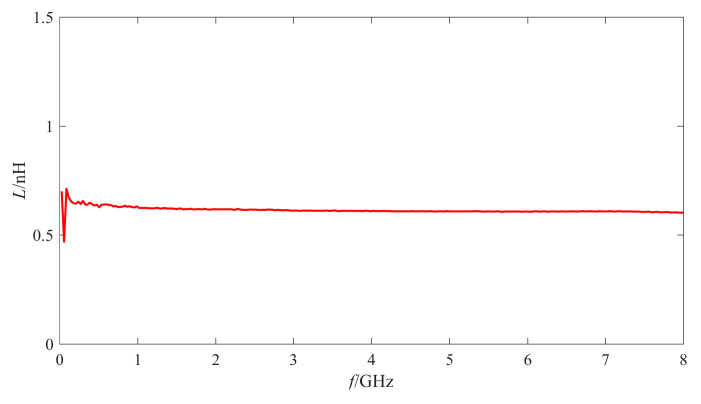
The inductance of one fabricated MEMS suspended inductor.

The de-embedded Q factor and the inductance of one inductor are shown in Figure 7 and Figure 8, respectively.

As shown in Figure 7, the Q factor of the fabricated MEMS suspended inductor is higher than 20 when the frequency is higher than 0.5 GHz and the maximum Q factor can reach 49 when the inductor works at 6 GHz. As shown in Figure 8, the inductance of the inductor is about 0.615 nH.

According to the measurement results, the fabricated MEMS suspended inductor samples have good RF performance. It can work in a wide frequency range and it can be used in 5–6 GHz voltage-controlled oscillator (VCO) and low noise amplifier (LNA) circuit, etc. In addition, several inductor samples fabricated on the same wafer were measured. The measurement results show that the maximum Q factors of the inductors are all in the range of 48–60 and the inductance values are all in the range of 0.61–0.63 nH, which also show that the inductors have good consistency.

## 3. Shock Test of MEMS Suspended Inductors

**Figure 9:**
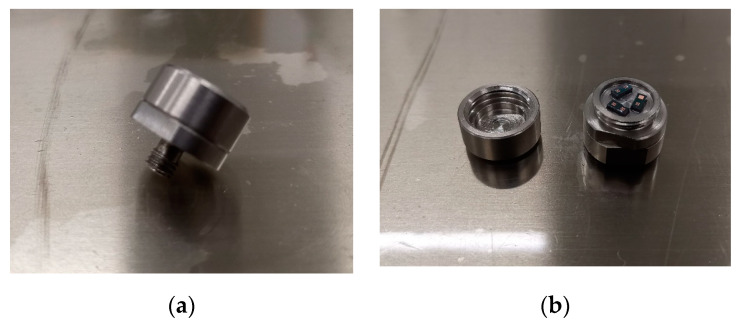
The shell with inductor dies for shock test. (**a**) The designed test shell structure. (**b**) The test shell with inductor dies.

In this study, an air cannon was chosen as the shock test equipment, because the volume of the air cannon was large and a single test can accommodate more inductor samples. Since the size of the MEMS suspended inductor samples were only in the micrometer level, it was necessary to adhere the inductors on the test shell for the shock test. The test shell designed for the test is shown in Figure 9.

Figure 9a shows the design of the shock test shell structure. The stud structure at the bottom of the shell was used to fix the shell on the air cannon. The material of the shell was made on high-strength stainless steel to avoid the deformation of the shell during the high-g shock test. The deformation of the shell may cause the internal inductor samples to be damaged or fall off. The wafer was divided into several dies and the size of each die was about 2 mm × 4 mm × 1 mm. Each die had one or two inductors on it. The dies were adhered to the test shells with epoxy resin adhesive and each shell contained three dies. The test shell with inductor dies is shown in Figure 9b. In order to ensure the consistency of the inductor samples for the shock test, all samples for the test were from the same wafer and the positions of samples on the wafer were close.

**Figure 10:**
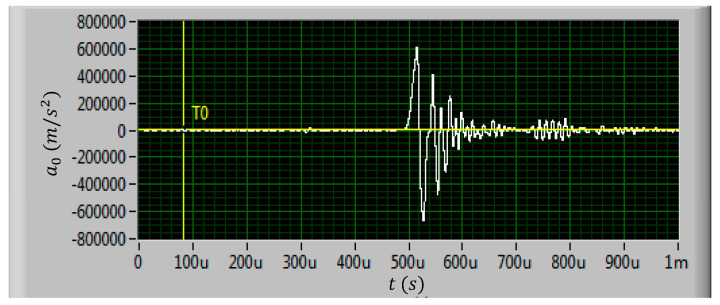
The measured 60,000 g shock load waveform.

**Figure 11:**
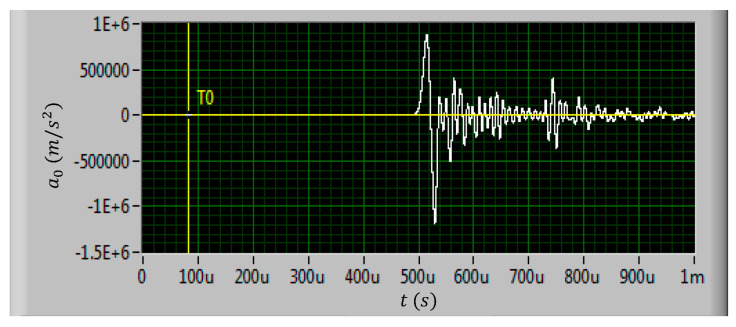
The measured 80,000 g shock load waveform.

**Figure 12:**
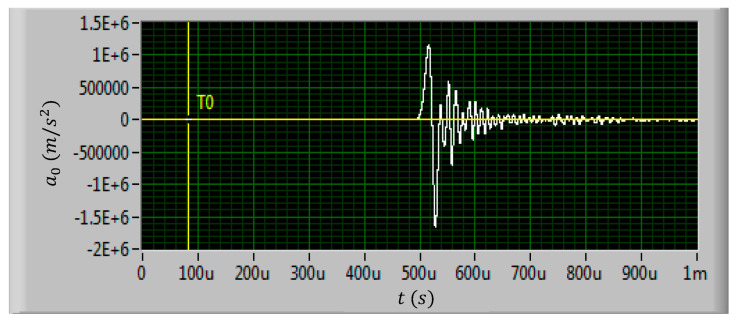
The measured 100,000 g shock load waveform.

A total of eight test shells were prepared for the shock test. The test shells were divided into three groups, and shock tests with three different kinds of shock loads were carried out. The amplitudes of the three kinds of shock loads were set to 60,000 g, 80,000 g, and 100,000 g, respectively. The shock load direction was applied perpendicular to the inductor coil plane as the normal direction of the coil plane is the shock sensitive direction of the inductor [18]. The shock load waveforms generated by the air cannon are shown in Figure 10, Figure 11 and Figure 12.

In actual shock test, it is difficult to ensure that the air cannon can generate standard half sine pulse waveform. It can be found in Figure 10, Figure 11 and Figure 12 that the actual shock waveform generated by the air cannon was an oscillation waveform. The acceleration peak values of the shock loads were about 611,782 m/s^2^, 870,936 m/s^2,^ and 1164,121 m/s^2^, respectively. The duration of the first peak were all about 20 μs and the total duration of the shock loads were all about 100 μs.

**Figure 13:**
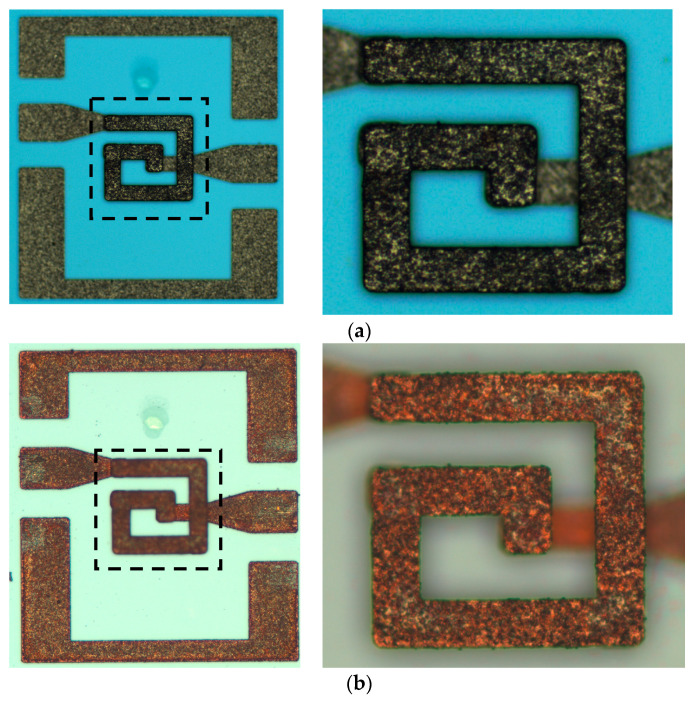
The intact inductor before and after 60,000 g shock test. (**a**) Before shock test; (**b**) after shock test.

**Figure 14:**
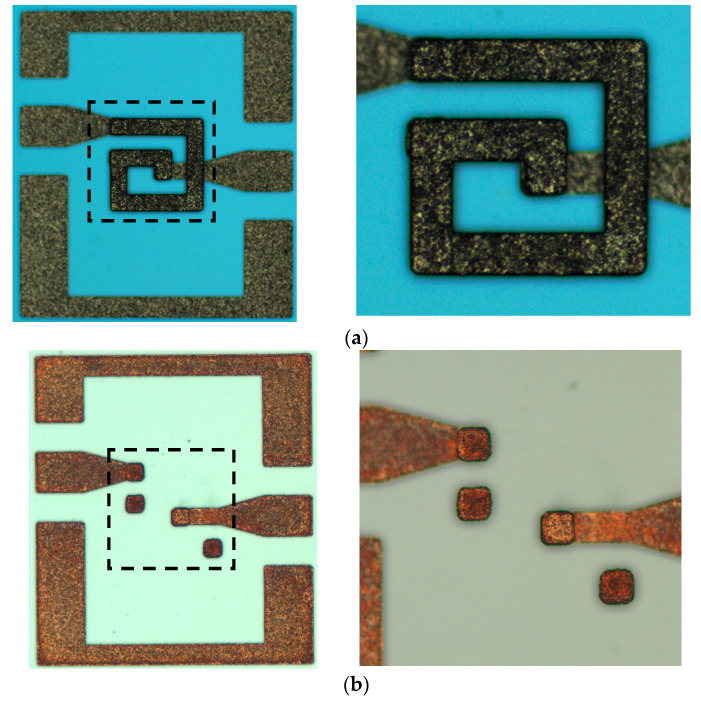
The failed inductor before and after 60,000 g shock test. (**a**) Before shock test; (**b**) after shock test.

After the shock test of eight shells was completed, the shocked MEMS suspended inductors were observed under microscope, compared with the photos before the shock test to observe the deformation and damage of each inductor structure. In this section, taking the inductors after 60,000 g shock test as an example, a group of comparison pictures of intact and failed inductors were given, as shown in Figure 13 and Figure 14.

By observing all failed MEMS suspended inductors after the shock test, it can be found that the failure mode of failed inductors was the same, which was the whole inductor coil falling off, as shown in Figure 14. The photos before and after the shock test of all of the intact inductors were compared, and no visible structural damage was observed. As the image is top view, it is difficult to show the deformation of the coil in the vertical direction along the plane of the coil. But it can be seen that the width and the total length of the coil wire were not significantly affected by the shock, so according to the π model, the elements of the parallel branch of the model are mainly affected by the suspension height of the inductor. In the next section, the influence of shock on the variation of the π model elements and the performance of the inductors will be analyzed.

The number of intact inductors after shock was counted and the results of shock test were listed in Table 1.

It can be found from Table 1 that 6 of 8 inductors used in the 60,000 g shock test remained intact and no visible damage was observed from them, such as coil falling off and fracture, and the intact rate was 75%. For the 80,000 g shock test, 3 of 10 inductors remained intact and the intact rate was only 30%. For the 100,000 g shock test, 5 of 14 inductors remained intact and the intact rate was only 35%. The results show that most of the inductor samples can bear the 60,000 g shock, but when the shock acceleration amplitude increased to 80,000 g or more, the intact rate of the inductor samples decreased significantly. Although the excellent mechanical properties of the inductor were verified by calculation in design stage, a number of inductors failed in the actual shock test, especially in the case of the high acceleration amplitude shock test.

It can be seen from Figure 10 to Figure 12 that the actual shock wave generated by the air cannon is an oscillation waveform, which is different from the ideal half sine wave. The actual waveform is first a full period sine wave with the set acceleration amplitude, followed by an oscillation with a decreasing amplitude. As the shock load direction is set to be upward along the normal direction of the inductor coil, when the first half sine period with positive acceleration was loaded on the MEMS suspended inductor structure, the inductor coil would vibrate downward, when the second half sine period with negative acceleration was loaded, the inductor coil would deform upward. No matter which direction the MEMS suspended inductor coil vibrates and deforms, the position where the coil is connected with the pillar of port 1 (see Figure 1) is the critical point as the maximum stress of the inductor occurs at this position. This position will first reach the yield strength or even tensile strength of the coil material, i.e., copper, and lead to plastic deformation and even fracture failure of the coil.

As Figure 14b shows, only coil detached from the pillars and the detaching area is smooth, which indicates that the adhesion between the seed layer and the top of the pillar layer is insufficient. MEMS suspended inductors were a layered structure, and they were fabricated by the layered electroplating process. The adhesion between the pillar layer and the coil layer can be affected by the electroplating process parameters and the presence of impurities such as residual photoresist on the contact surface of the layers, these unavoidable defects in the process will lead to poor adhesion between the layers. In addition, there was a Cu/Cr sputtered seed layer between the pillar layer and coil layer, the mechanical properties and the residual stress of the seed layer were directly related to the magnetron sputtering parameters. The interlayer stress between the electroplated copper coil layer and the magnetron sputtered seed layer, which was difficult to avoid, was one of the main reasons that the mechanical properties was worse than the theoretical analysis.

In conclusion, considering the actual shock waveform and the inductor fabrication process, when the positive half sine wave was loaded to the inductor, the inductor coil vibrated downward and the maximum compressive stress occurred at the position where the coil was connected with the pillar of port 1. When the stress exceeded the yield stress of copper, plastic deformation occurred to the coil and the RF performance of the inductor would vary permanently. When the half sine wave in negative direction was loaded to the inductor, the coil vibrated upward and the maximum tensile stress also occurred at the same position. When the stress exceeded the bonding force between layers, the coil would tear apart from the pillars. Therefore, the failure mode of the inductors in the shock test was the whole inductor coil falling off.

## 4. Results and Discussion

**Figure 15:**
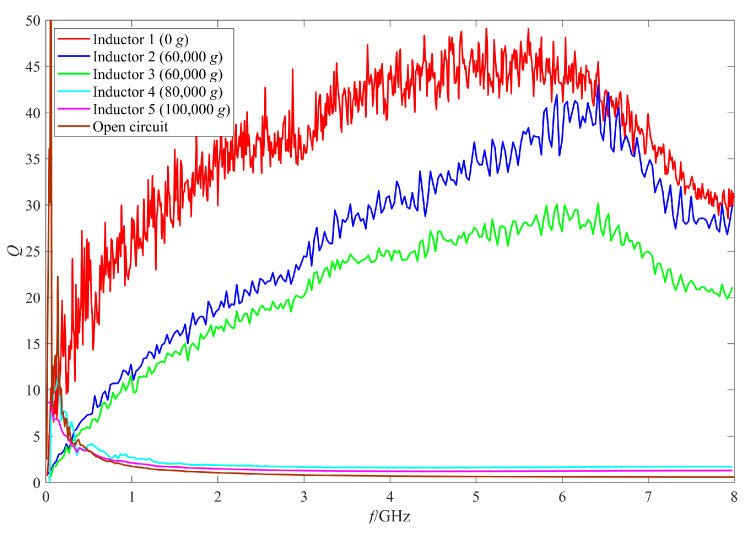
The Q factors of the inductors after shock test with different shock amplitude.

**Figure 16:**
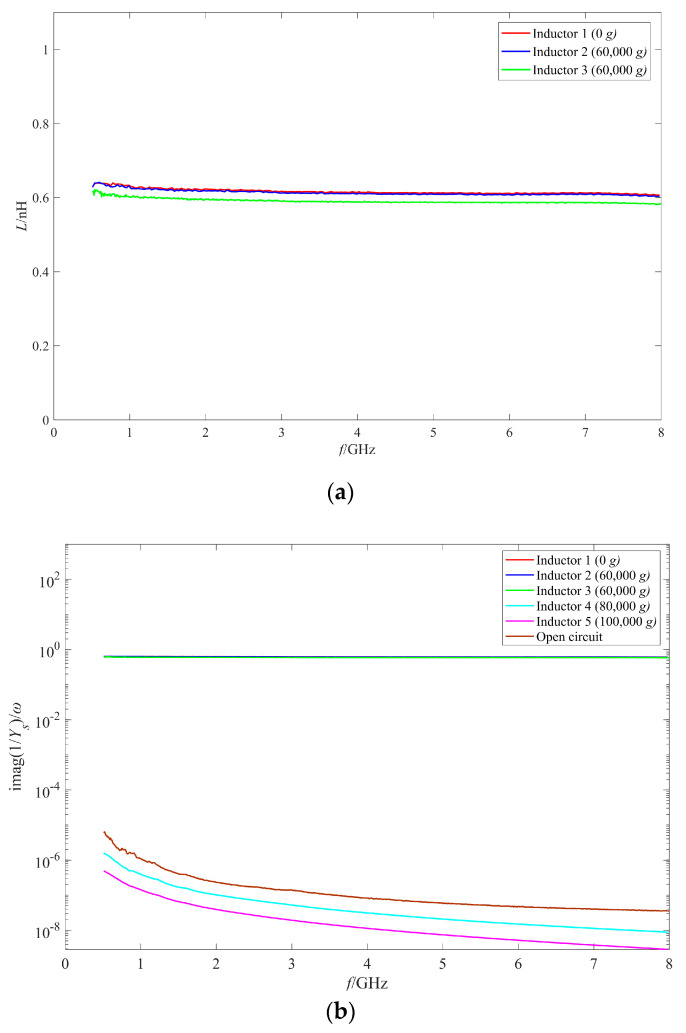
The inductance values of the inductors after shock test with different shock amplitude. (**a**) The inductance values of No.1–3 inductors; (**b**) The inductance values of No.1–5 inductors.

The RF performance of the intact inductors after the shock test were measured by an Agilent N5224a (Agilent, Palo Alto, CA, USA) vector network analyzer and a Cascade probe station. The Q factor and the inductance value of the intact inductors shocked by 60,000 g, 80,000 g, and 100,000 g are shown in Figure 15 and Figure 16 respectively. For the convenience of comparison, the measurement results of the unshocked inductor and the open circuit structure (no coil and only ground ring) are also given in the figures. The unshocked inductor, the two inductors shocked by 60,000 g, the inductor shocked by 80,000 g, and 100,000 g are numbered as 1–5.

Figure 15 shows that the Q values of the two inductors, No.2 and No.3, which were subjected to 60,000 g shock, decreased in different degrees. The maximum Q factor of No.2 inductor decreased from 49.08 at 6 GHz to 42.96 at 6.3 GHz. Although the decrease of the maximum Q factor was small, the Q factor of the inductor No.2 at lower frequencies (below 5 GHz) decreased more significantly. The maximum Q factor of No.3 inductor decreased more significantly from 49.08 to 30.15 at 6 GHz and the Q factor of No.3 inductor decreased significantly in the whole 0–8 GHz frequency band. No.1–No.3 inductors all reach the maximum Q factor at the frequency range around 5.5–6 GHz, so the frequency at which the inductor reaches the maximum Q factor did not vary much after the shock test. Figure 16 shows that the inductance values of the two inductors subjected to 60,000 g shock did not vary much compared with that of the unshocked inductor. The inductance of No.2 inductor remained at 0.61 nH, while the inductance of No.3 inductor decreased slightly to 0.59 nH, only 0.02 nH. So of the two inductors subjected to 60,000 g shock, the coil of No.2 inductor experienced elastic deformation, the coil deformation recovered after the load unloading, while the coil of No.3 inductor experienced slight plastic deformation.

Figure 15 and Figure 16 also show that the Q factors and inductance values of No.4 and No.5 inductors shocked by 80,000 g and 100,000 g were abnormal, which indicated that although no visible structure damage was found on the inductors, they still failed and did not work properly. Comparing the measured results of No.4 and No.5 inductors with the open circuit structure, it can be found that the Q factors and the inductance values of the two inductors were similar to the measured results of the open circuit structure. In addition, the measured results of other intact inductors shocked by 80,000 g and 100,000 g were also similar to those of No.4 and No.5 inductors in Figure 15 and Figure 16. So, it can be concluded that the reason for the abnormal results was that an open circuit occurred to the inductor.

**Figure 17:**
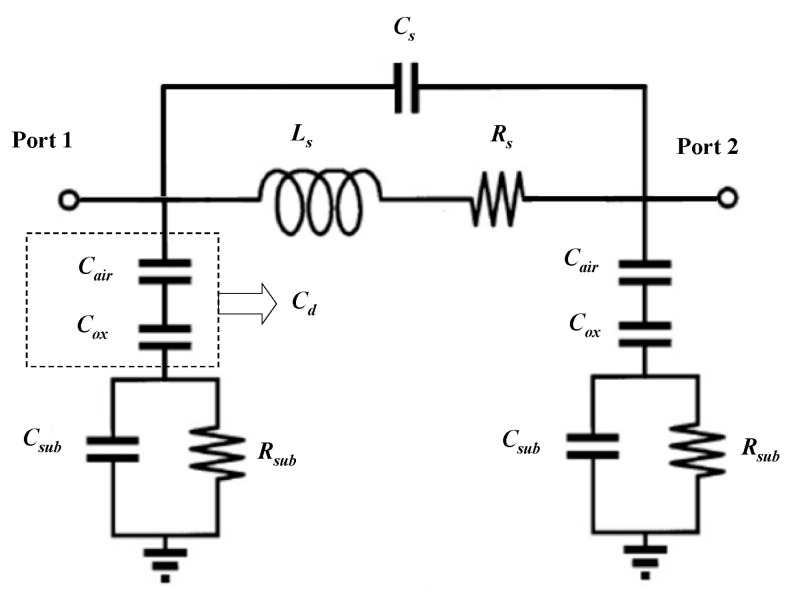
The π lumped-element equivalent model of MEMS suspended inductor.

In this section, by extracting the π lumped-element equivalent model parameters using the measured S parameters, the RF performance variation mechanism of the intact inductors after shock and the failure mechanism of the failed inductors were analyzed. The π lumped-element equivalent model was proposed by Yue et al. [24,25,26] and the schematic of the model is shown in Figure 17. 

**Figure 18:**
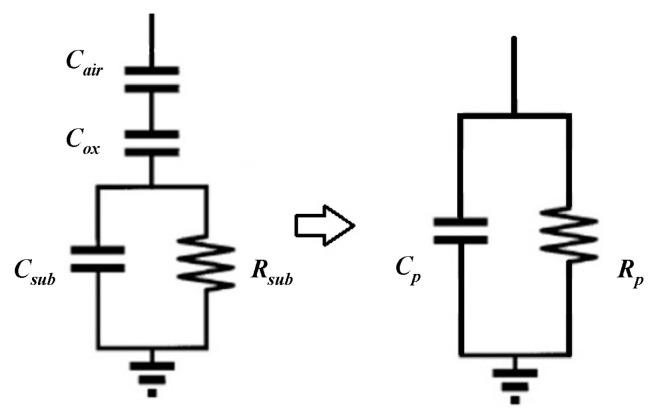
The simplified parallel branch of the π model.

The equivalent circuit consists of a series branch and two parallel branches. The series branch consists of three elements: the series inductance $L_{s}$, the series resistance $R_{s},$ and the series capacitance $C_{s}$. Each parallel branch consists of three elements: the dielectric layer capacitance $C_{d}$, the substrate parasitic resistance $R_{sub},$ and the substrate parasitic capacitance $C_{sub}$. To characterize the losses caused by the substrate containing air layer and insulating layer more clearly, the parallel branch is simplified to a resistor–capacitor parallel circuit as Figure 18 shows.

The resistance $R_{p}$ and the capacitance $C_{p}$ represent the substrate loss and the overall substrate parasitic capacitance respectively.

### 4.1. Analysis of the RF Performance Variation Mechanism of the Intact Inductors

**Figure 19:**
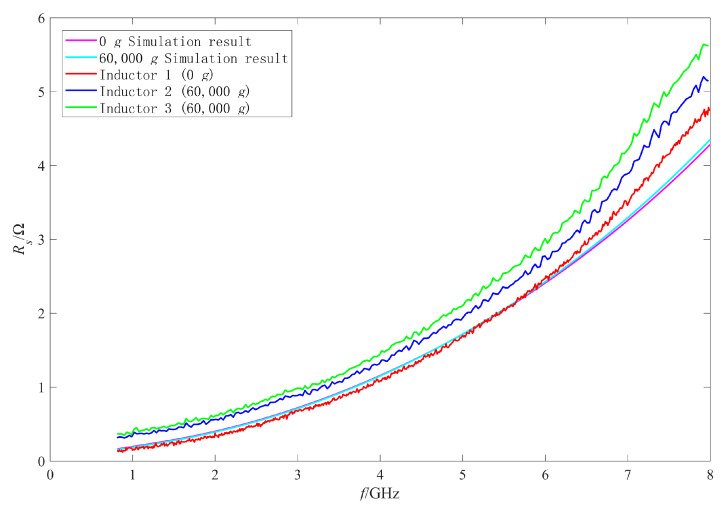
The measured and simulated $R_{s}$ of the intact inductors before and after shock test.

**Figure 20:**
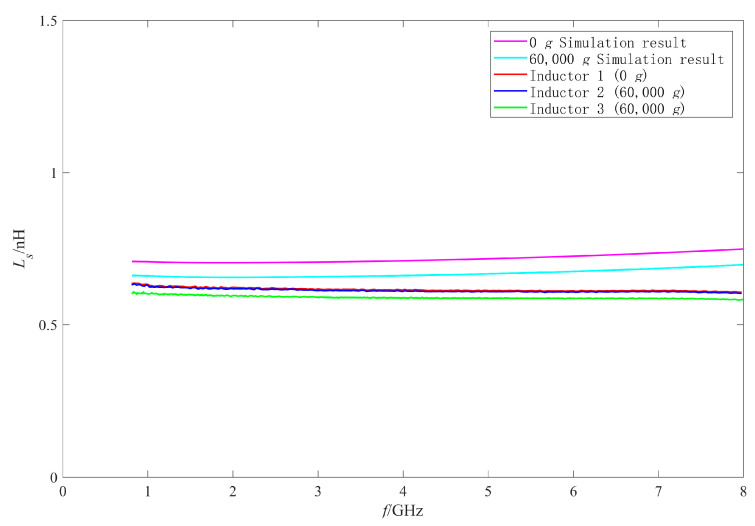
The measured and simulated $L_{s}$ of the intact inductors before and after shock test.

**Figure 21:**
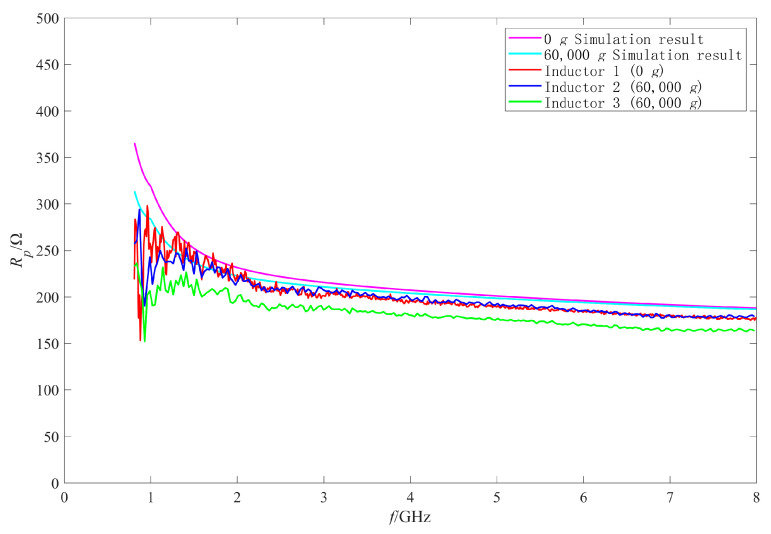
The measured and simulated $R_{p}$ of the intact inductors before and after shock test.

**Figure 22:**
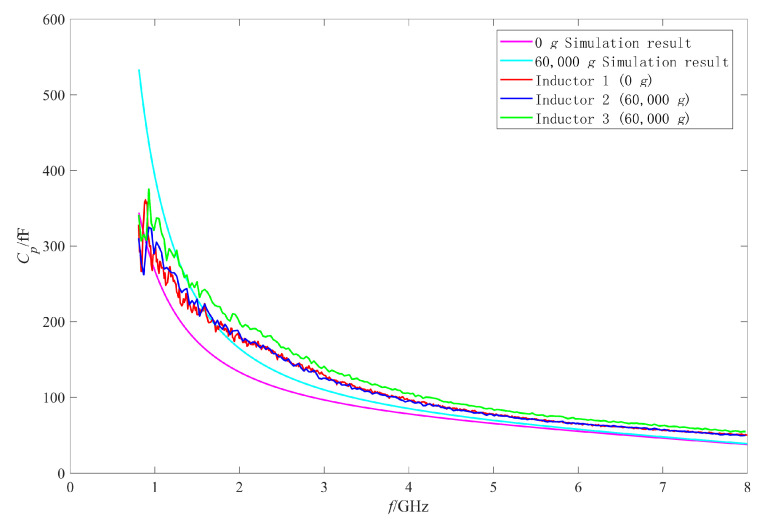
The measured and simulated $C_{p}$ of the intact inductors before and after shock test.

Firstly, the RF performance variation mechanism of the intact inductors was analyzed. The π model parameters were extracted with the method in [25,27,28]. Figure 19, Figure 20, Figure 21 and Figure 22 show the measured results of the series resistance $R_{s}$, series inductance $L_{s}$, substrate parasitic resistance $R_{p},$ and the substrate parasitic capacitance $C_{p}$ of the inductors subjected to 60,000 g shock load. Table 2 shows the series capacitance measured results of the inductors. For comparison, the simulated results of the model parameters of the inductor subjected to 60,000 g shock load at the moment of maximum deformation, the measured and the simulated results of the model parameters of the unshocked inductor were also given in the table and figures.

The RF performance variation of No.2 inductor is analyzed firstly. According to the measured results in Figure 20, Figure 21 and Figure 22 and Table 2, the series inductance $L_{s}$, series capacitance $C_{s}$, substrate parasitic resistance $R_{p},$ and the substrate parasitic capacitance $C_{p}$ of No.2 inductor had no obvious variation compared with No.1 inductor which was not shocked. This means that no plastic deformation occurred to the coil of No.2 inductor when it was subjected to 60,000 g shock and its suspension height did not decrease. However, it can be found from Figure 19 that the series resistance $R_{s}$ of No.2 inductor increased compared with No.1 inductor in the whole 0–8 GHz frequency band, from 4.75 Ω to 5.14 Ω at 8 GHz (increased by 8.2%) and from 0.15 Ω to 0.33 Ω at 1 GHz (increased by 120%). The series resistance varied more significantly at low frequency, so the Q factor of No.2 inductor in Figure 15 decreased more obviously at low frequency, and did not vary significantly at high frequency. Therefore, the variation of Q factor of No.2 inductor was mainly affected by the variation of the series resistance rather than the increase of substrate loss caused by the decrease of the suspension height after shock test. According to [29], the series resistance $R_{s}$ of the inductor coil considering the skin effect and proximity effect can be expressed as:

(3) $$R_{s}=R_{\mathrm{dc}}\left[1+\frac{1}{10}{\left(\frac{\omega }{\omega_{\mathrm{crit}}}\right)}^{2}\right]$$

where ω is the angular frequency, $R_{\mathrm{dc}}$ is the coil’s DC resistance, and $\omega_{\mathrm{crit}}$ is the frequency that the proximity effect begins to become significant. $\omega_{\mathrm{crit}}$ is related to the wire width, spacing, and the sheet resistance of the wire. The series resistance of the inductor increased at both high and low frequencies, it indicates that the DC resistance of the inductor coil increased. High-g shock would lead to the temperature variation of the MEMS devices, according to reference [30], high-g shock and the temperature variation of the material would affect the material properties, such as the surface roughness, the uniformity and the consistency of the material. The material properties variation would affect the resistivity of the material and cause the variation of the DC resistance of the inductor coil. It is considered that this is the main reason that the series resistance of the inductor varied in the whole test frequency band after shock test. As the inductor performance variation caused by coil deformation is mainly studied in this paper, we will continue our study further on the influence of temperature on the inductor performance.

Then the performance variation of No.3 inductor, which was also subjected to 60,000 g shock, is analyzed. Figure 15 shows that the Q factor of No.3 inductor varied significantly in the whole test frequency band. It can be found from Table 2, Figure 20, Figure 21 and Figure 22 that $C_{s}$ of No.3 inductor increased by about 0.2 fF, $L_{s}$ decreased by 0.023 nH (only 3.7% of the No.1 inductor $L_{s}$), $R_{p}$ decreased from 255 Ω to 212 Ω at low frequency 1 GHz and from 179 Ω to 163 Ω at high frequency 8 GHz, and $C_{p}$ increased from 290 fF to 337 fF at low frequency 1 GHz and from 50 fF to 55 fF at high frequency 8 GHz. As the comparison photos of inductors before and after the shock test in Figure 13 shows that the line width, spacing, the total length of the coil, and the properties of the substrate material were not significantly affected by the shock. The increase of $C_{s}$, $C_{p}$ and the decrease of $R_{p}$ were mainly due to the increase of the dielectric layer capacitance between the inductor coil and the substrate, which indicates that plastic deformation occurred to the No.3 inductor coil and the inductor suspension height decreased. The variation of $C_{s}$, $R_{p},$ and $C_{p}$ indicated that the substrate loss and the loss caused by electric energy stored by the inductor increased when the inductor was shocked and the suspension height decreased.

Comparing the measured results of $C_{s}$, $R_{p,}$ and $C_{p}$ of No.3 inductor with the simulated results, it can be found that the measured variations are slightly larger than the simulated variations before and after the shock. This means that the inductor coil deformation is slightly larger than the calculated results because the actual shock load was an oscillation waveform and its amplitude was greater than 60,000 g. 

In addition, Figure 19 shows that $R_{s}$ of No.3 inductor increased from 0.15 Ω to 0.36 Ω at low frequency of 1 GHz and from 4.75 Ω to 5.60 Ω at high frequency of 8 GHz. This means that $R_{s}$ of No.3 inductor also increased in the whole test frequency band and the ohmic loss of the inductor increased. Therefore, the increase of ohmic loss, substrate loss, and loss caused by electric energy stored by the inductor lead to the significant decrease in Q factor of No.3 inductor in the whole test frequency band.

### 4.2. Analysis of the Failure Mechanism of the Failed Inductors

**Figure 23:**
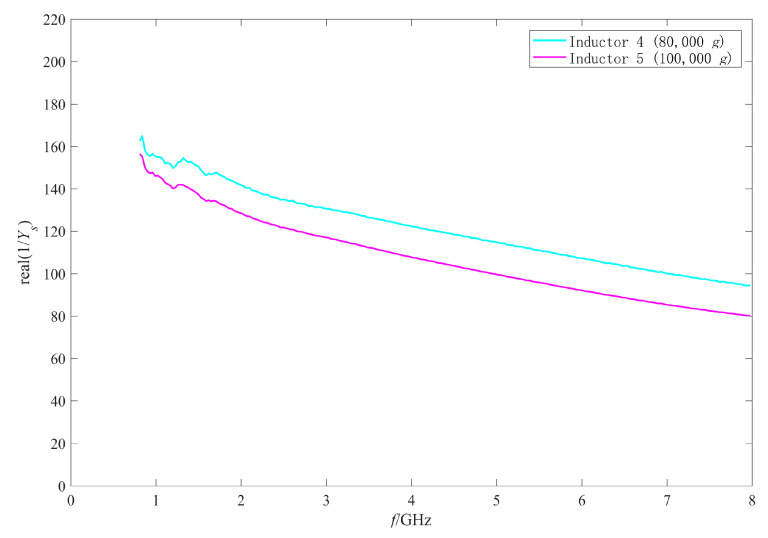
The real part of $Y_{s}$ of the failed inductors after shock test.

**Figure 24:**
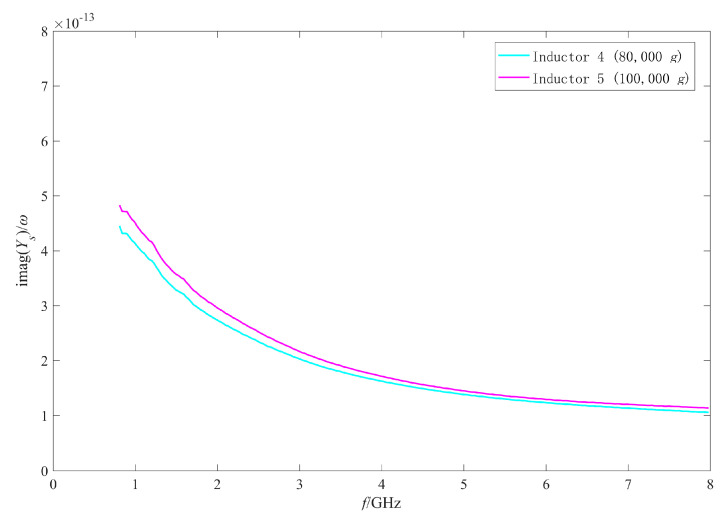
The imaginary part of $Y_{s}$ of the failed inductors after shock test.

**Figure 25:**
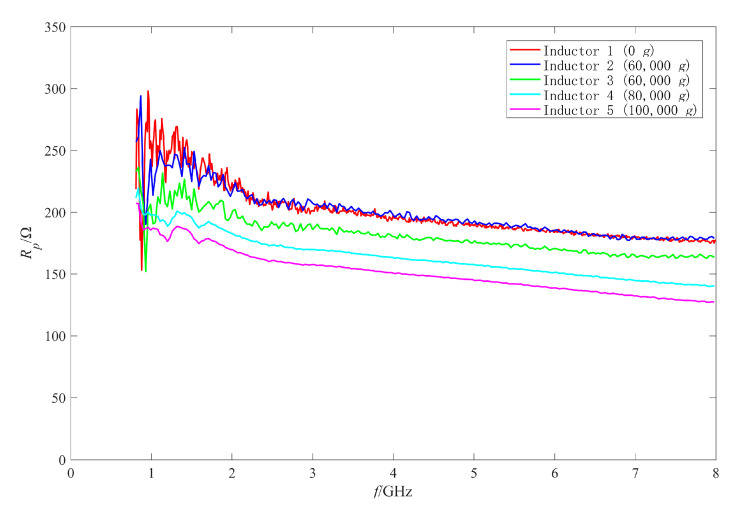
$R_{p}$ of the failed inductors after shock test.

**Figure 26:**
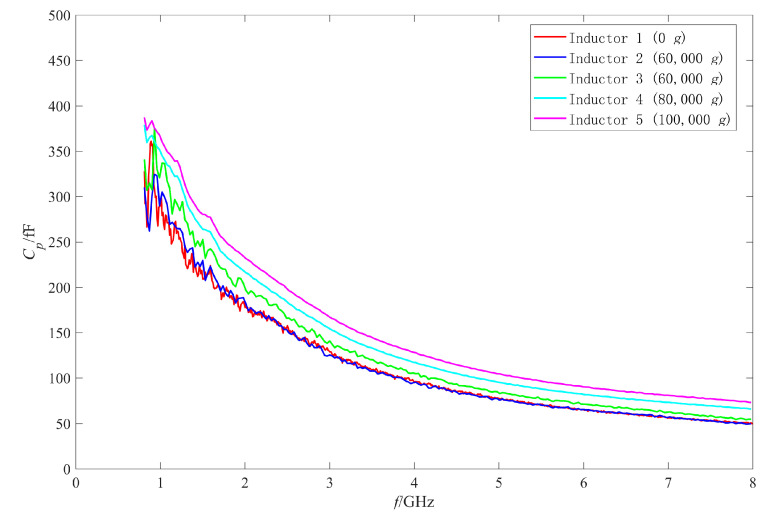
$C_{p}$ of the failed inductors after shock test.

As the measured results of the inductors shocked by 80,000 g and 100,000 g were abnormal and similar to those of open circuit structure, it was considered that the reason for the abnormal results was the occurrence of an open circuit to the inductors. In this section, the π model parameters were extracted to analyze the failure mechanism of the failed inductors. Figure 23 and Figure 24 show the extracted results of the real part and the imaginary part of the admittance parameter of the series branch (i.e., $Y_{s}$) of the failed inductors, respectively. Figure 25 and Figure 26 show the extracted results of the substrate parasitic resistance $R_{p}$ and the substrate parasitic capacitance $C_{p}$ of the failed inductors, respectively. The extracted results of $R_{p}$ and $C_{p}$ of the unshocked inductor and inductors shocked by 60,000 g are also given in Figure 25 and Figure 26.

When the inductor is open circuit, the series branch of the π model is open circuit, the extracted results of the real part and the imaginary part of $Y_{s}$ are actually the substrate parasitic resistance and the substrate parasitic capacitance of the inductor, respectively. Comparing the parameter extracted results of No.4 inductor (shocked by 80,000 g) and No.5 inductor (shocked by 100,000 g) in Figure 23, Figure 24, Figure 25 and Figure 26, it can be found that the values of $\mathrm{real}(1/Y_{s})$ and $R_{p}$ are close, $\mathrm{imag}(Y_{s})/\omega$ and $C_{p}$ are close. This further indicates that the series branch of the inductors shocked by 80,000 g and 100,000 g were open circuit. The DC resistance of the no visible damage failed inductor device with abnormal RF behavior is then measured with a Cascade probe station. The results show that the DC resistance of the device is 9.95 Ω, which is nearly 200 times and much higher than that of the inductor before the shock test (about 0.05 Ω by calculating with the structural parameters of the coil). This also indicates that the coil of the failed inductor is in poor contact with the ports after the shock test.

Although the coil did not fall off, the connection between the coil and the pillar was still prone to be disconnected for the following reasons: first, the shock load waveform generated by air cannon was an oscillation waveform rather than an ideal half sine waveform and the pillars cannot support and restrain the inductor coil when it was shocked and vibrated upward; second, the maximum stress appears at the critical position where the coil is connected with the pillar of port 1 when the inductor is shocked; third, the adhesion between the inductor coil and the pillars is worse than the theoretical analysis due to the influence of the manufacturing process.

Figure 25 shows that the extracted results of $R_{p}$ of No.4 and No.5 inductor decreased compared with those of unshocked inductor. Figure 26 shows that the extracted results of $C_{p}$ of the shocked inductors increased. The higher the shock load amplitude is, the more serious the variation of $R_{p}$ and $C_{p}$ after the shock test is. Although the series branch extracted results show that the inductors subjected to the shock load with higher than 80,000 g acceleration amplitude had an open circuit, there was still a partial connection between the coil and the substrate due to the existence of auxiliary pillars and only one of the two ports had an open circuit. As the shock did not affect the area of the inductor coil and the properties of the substrate material, the factor affecting the substrate parasitic effect was still the dielectric layer capacitance, which depended on the suspension height of the inductor. Therefore, plastic deformation also occurred to the No.4 and No.5 inductor. The higher the shock acceleration amplitude is, the more serious the deformation is.

In conclusion, the main reason for the RF performance variation is the decrease of the suspension height caused by plastic deformation of the inductor coil. The decrease of the suspension height leads to the increase of the substrate loss and the loss caused by the electric energy stored by the inductor, and these losses make the RF performance of the inductor become worse. In addition, it can be found from the measured results that the shock not only caused deformation of the inductor structure, but also affected the properties of the coil material, which resulted in the variation of the resistance of the coil and the increase of ohmic loss. By analyzing the measured results of the intact inductors without fracture or coil falling off after shock, it can be found that some of the inductors shocked by 60,000 g shock load had plastic deformation and others did not. All inductors shocked by shock load with 80,000 g and above acceleration amplitude had open circuit and all of them had plastic deformation. The results indicate that 60,000 g is the critical shock acceleration amplitude for plastic deformation of the inductor and the actual yield strength of the inductor is about 42 MPa.

## 5. Conclusions

In this paper, a kind of MEMS suspended inductor with excellent RF and mechanical performance is designed and fabricated, the RF performance variation and failure mechanism of the MEMS suspended inductor under high-g shock of which acceleration amplitude is as high as tens of thousands of g are studied with this kind of inductor. The fabrication process of the inductor with double auxiliary pillars is given and the air cannon shock tests are carried out. The RF performance variation and failure mechanism are analyzed by extracting the values of each π model parameters of the inductors after the shock test. The results show that the quality factor and the inductance value of the inductors decrease in different degrees after shock. The performance variation is mainly due to the variation of the substrate parasitic effect caused by the deformation of the inductor coil and also related to the variation of the properties of the coil material affected by high-g shock. The increase of the substrate parasitic capacitance leads to the increase of the electric field energy stored and the decrease of the substrate parasitic resistance leads to the increase of substrate loss. The increase of these losses leads to the decrease of the quality factor and the inductance value of the inductors after shock. The failure modes of the failed inductors after shock are coil falling off or open circuit, which is different from the results of the shock test with low acceleration amplitude. The oscillation waveform of shock load generated by air cannon, the poor interlayer bonding caused by fabrication process parameters and the maximum stress occurring at the interlayer joint lead to the inductors fail more easily than the theoretical analysis. In addition, the results indicate that the actual yield strength and the critical shock acceleration amplitude for plastic deformation of the inductor are about 42 MPa and 60,000 g, respectively. Through the research of this paper, it is found that the high-g shock will not only lead to the deformation and then the performance variation of the inductor, but also affect the material properties. Besides, the failure mode of the inductor under high-g shock is also different from that under shock with low acceleration amplitude, so the influence of the high-g shock and shock with low acceleration amplitude on MEMS suspended inductors needs to be analyzed separately. According to the results, high-g shock and temperature variation during shock will affect the material properties of the coil, and high-g shock can easily lead to the interlayer tearing of the inductor. Therefore, further studies are needed on the influence of high-g shock and temperature on the electrical properties of electroplating copper film and the fabrication process optimizing of inductors through a number of fabrication process experiments to improve the interlayer adhesion and the mechanical properties of the inductors.

## Figures and Tables

**Table 1:** The results of shock test.

Shock Amplitude	Shell Number	Number of Inductor Samples	Number of Intact Inductors	Intact Rate of the Inductors After Shock
100,000 g	1 2 3	5 4 5	0 3 2	35%
80,000 g	4 5 6	3 3 4	1 1 1	30%
60,000 g	7 8	4 4	3 3	75%

**Table 2:** The measured and simulated $C_{s}$ of the intact inductors before and after shock test.

Shock Amplitude	The Measured $C_{s}$/fF	The Simulated $C_{s}$/fF
0 g	1.5275 (Inductor 1)	1.4722
60,000 g	1.5513 (Inductor 2)	1.5497
	1.7230 (Inductor 3)	

## References

[1] Shi J., Yin W.-Y., Liao H., Mao J.-F. (2006). The enhancement of Q factor for patterned ground shield inductors at high temperatures. IEEE Trans. Magn..

[2] Lin Y.-S., Chen C.-Z., Liang H.-B., Chen C.-C. (2007). High-performance on-chip transformers with partial polysilicon patterned ground shields (PGS). IEEE Trans. Electron Devices.

[3] Wang Y.F., Liao X.P., Huang Q.A. (2004). Research and Progress of RF MEMS Inductors. Chin. J. Electron. Dev..

[4] Yoon J.B., Han C.H., Yoon E., Kim C.K. Monolithic high-Q overhang inductors fabricated on silicon and glass substrates. Proceedings of the IEEE International Electron Devices Meeting.

[5] Yoon J.B., Han C.H., Yoon E., Kim C.K. High-performance three-dimensional on-chip inductors fabricated by novel micromachining technology for RF MMIC. Proceedings of the IEEE international Microwave Symposium Digest.

[6] Ding Y., Liu Z., Liu L., Li Z. (2003). A surface micromachining process for suspended RF-MEMS applications using porous silicon. Microsyst. Technol..

[7] Wang X.N., Zhao X.L., Zhou Y., Dai X.H., Cai B.C. (2004). Fabrication and performance of a novel suspended RF spiral inductor. IEEE Trans. Electron Devices.

[8] Tai C.M., Liao C.N. (2007). Multilevel suspended thin-film inductors on silicon wafers. IEEE Trans. Electron Devices.

[9] Zheng T., Xu G., Luo L. (2017). High performance suspended spiral inductor and band-pass filter by wafer level packaging technology. Microsyst. Technol..

[10] Li L., Ma K., Mou S. A Novel High Q Inductor Based on Double-sided Substrate Integrated Suspended Line Technology with Patterned Substrate. Proceedings of the IEEE MTT-S International Microwave Symposium (IMS).

[11] Stojanović G., Živanov L., Damnjanović M. (2006). Novel efficient methods for inductance calculation of meander inductor. COMPEL Int. J. Comput. Math. Electr. Eng..

[12] Wang T.P., Li Z.W., Tsai H.Y. (2013). Performance Improvement of a 0.18-μm CMOS Microwave Amplifier Using Micromachined Suspended Inductors: Theory and Experiment. IEEE Trans. Electron Devices.

[13] Park E.-C., Choi Y.-S., Yoon J.-B., Hong S., Yoon E. (2003). Fully integrated low phase-noise VCOs with on-chip MEMS inductors. IEEE Trans. Microw. Theory Tech..

[14] Mohammadi A., Karmakar N.C. High Quality MEMS Inductors for Chipless RFID in Harsh Environment. Proceedings of the 2016 IEEE 2nd Australian Microwave Symposium.

[15] Brown T.G., Davis B., Hepner D., Faust J., Myers C., Muller C., Harkins T., Holis M., Placzankis B. (2001). Strap-down microelectromechanical (MEMS) sensors for high-g munition applications. IEEE Trans. Magn..

[16] Le H.T., Mizushima I., Nour Y., Torben Tang P., Knott A., Ouyang Z., Jensen F., Han A. (2017). Fabrication of 3D air-core MEMS inductors for very-high-frequency power conversions. Microsyst. Nanoeng..

[17] Lin J.W., Chen C.C., Cheng Y.T. (2005). A robust high-Q micromachined RF inductor for RFIC applications. IEEE Trans. Electron Devices.

[18] Chiu Y., Hong H.C., Wu P.C. (2013). Development and Characterization of a CMOS-MEMS Accelerometer with Differential LC-Tank Oscillators. J. Microelectromech. Syst..

[19] Wu J.C., Zaghloul M.E. (2008). CMOS Micromachined Inductors with Structure Supports for RF Mixer Matching Networks. IEEE Electron Devices Lett..

[20] Li Y.Y., Xu L.X., Li J.H. (2019). Analysis of the Performance Variation Mechanism of MEMS Suspended Inductors under Mechanical Shock. Micromachines.

[21] Srikar V.T., Senturia S.D. (2002). The reliability of microelectromechanical systems (MEMS) in shock environments. J. Microelectromech. Syst..

[22] Xu L., Li Y., Li J., Lu C. (2018). Mechanical Response of MEMS Inductor with Auxiliary Pillar under High-g Shock. Micromachines.

[23] Hikmat O.F., Ali M.S.M. (2017). RF MEMS Inductors and Their Applications—A Review. J. Microelectromech. Syst..

[24] Yue C.P., Ryu C., Lau J., Lee T.H., Wong S.S. A Psysical Model for Planar Spiral Inductors on Silicon. Proceedings of the International Electron Devices Meeting Technical Digest.

[25] Yue C.P., Wong S.S. (1998). On-Chip Spiral Inductors with Patterned Ground Shields for Si-Based RF IC’s. IEEE J. Solid State Circuits.

[26] Yue C.P., Wong S.S. (2000). Psysical Modeling of Spiral Inductors on Silicon. IEEE Trans. Electron Devices.

[27] Huang F.Y., Jiang N., Bian E.L. (2005). Modeling of single-π equivalent circuit for on-chip spiral inductors. Solid State Electron..

[28] Huang F.Y., Lu J.X., Jiang D.M., Wang X.C., Jiang N. (2006). A novel analytical approach to parameter extraction for on-chip spiral inductors taking into account high-order parasitic effect. Solid State Electron..

[29] Kuhn W.B., Ibrahim N.M. (2001). Analysis of Current Crowding Effects in Multiturn Spiral Inductors. IEEE Trans. Microw. Theory Tech..

[30] Liu X.D., Shang D.G., Liu H., Xu M.H., Xu J., Liu T.L. (2012). Study of Fatigue Property for Electroplated Copper Thin Film by Pulsed Laser Shock Peening. J. Mech. Strength.

